# A State Society for Eugenics

**Published:** 1913-07

**Authors:** 


### Department of Eugenics.

Conducted by Dr. Malone Duggan and Dr. Theo. Y. Hull.

### THE TEXAS STATE SOCIETY OF SOCIAL HYGIENE.

Headquarters: San Antonio, Texas.

DR. MALONE DUGGAN, President, San Antonio.
DR. F. E. DANIEL, 1st Vice President, Austin.
PROF. A. CASWELL ELLIS, 2d Vice President, Austin.
MRS. ELI HERTZBERG, 3d Vice President, San Antonio.
DR. THEO. Y. HULL, Secretary, San Antonio.
MR. W. L. EVANS, Treasurer, San Antonio.

EXECUTIVE COMMITTEE.

| | | |
|---|---|---|
| Dr. Malone Duggan, Chm. | Hon. J. C. Willacy. | Dr. W. A. King. |
| Dr. Theo. Y. Hull, Sec'y. | Mr. W. L. Evans. | Dr. J. W. Torbett. |
| Dr. W. F. McCaleb. | Dr. Frank D. Boyd. | Dr. F. C. Walsh. |
| Hon. C. H. Jenkins. | Dr. J. R. Nichols. | Dr. Joe Reuss. |
| Rev. A. W. S. Garden. | Dr. J. M. McCutchan. | Dr. Frank Paschal. |

### A State Society for Eugenics.

The eugenics movement is worldwide. Since the organization of a society at Washington, backed by the presence, approval and support of Mrs. Wilson, Mrs. Bryan, Mrs. John Hays Hammond and others of the elite, the subject has received a fresh impetus. A national society will be organized and every State will organize. Texas, always to the front in advocating reforms, and already in the field with its Society of Social Hygiene, has acted.

The Social Hygiene Society, at a meeting held in Austin July 3, was reorganized, and the name changed to "The Texas State Society of Social Hygiene and Eugenics." Officers were elected and an executive committee of twenty-five prominent men and women from all over the State was appointed, as well as "an active committee of three"—the president, secretary and treasurer, and one to be named. Details will have to be deferred till next issue, pending certain fundamental arrangements.

